# A network approach to understanding occupational psychological distress: linking depression, anxiety, and burnout among Chinese healthcare professionals

**DOI:** 10.3389/fpsyg.2024.1474523

**Published:** 2024-12-18

**Authors:** Cui Yang, Yao Chen, Xuelian Wang, Ping Xu, Juan Song, Lu Yang, Yue Fu

**Affiliations:** ^1^Department of Emergency, Zigong Fourth People’s Hospital, Zigong, Sichuan, China; ^2^Department of Nursing, Zigong Fourth People’s Hospital, Zigong, Sichuan, China; ^3^School of Psychology, Chengdu Medical College, Chengdu, Sichuan, China; ^4^School of Nursing, Chengdu Medical College, Chengdu, Sichuan, China

**Keywords:** job burnout, anxiety, depression, medical worker, network analysis

## Abstract

**Objectives:**

As a population at high risk for psychological distress, healthcare workers typically experience varying degrees of anxiety, depression, and burnout. Studies have found that depression and anxiety have a negative impact on the mental health domain of burnout in healthcare workers. However, little is known about the symptom-to-symptom interactions between these psychological outcomes. This study aims to elucidate the characteristics of depression, anxiety, and burnout networks among healthcare workers.

**Methods:**

We recruited 846 healthcare workers from March to April 2023 from three hospitals. A total of 826 healthcare workers completed the General Information Scale, the 16-item Quick Inventory of Depressive Symptomatology–Self-Report (QIDS-SR16), the Self-rating Anxiety Scale (SAS), and the Burnout Clinical Subtype Questionnaire (BCSQ)-36. The network models were constructed using network analysis. The expected influence and the bridge expected influence of nodes were calculated. The stability and accuracy of the network was assessed.

**Results:**

The results showed that the core symptoms in the symptom network mainly included QIDS8 (Energy/Fatigability), SAS3 (Easily upset or frightened), SAS11 (Dizzy), SAS8 (Tiredness), SAS10 (Tachycardia) and BCSQ3 (Worn-out), and the key nodes connecting these symptoms were QIDS2 (Sad mood), SAS20 (Have nightmares), BCSQ3 (Worn-out), SAS8 (Tiredness), QIDS8 (Energy/Fatigability), QIDS4 (Concentration/decision-making) and SAS4 (Madness).

**Conclusion:**

Unique pathways of association between burnout, depression, and anxiety were found to exist. Interventions targeting core symptoms can maximize the improvement of depression, anxiety, and burnout, provide a deeper understanding of the relationship between the three conditions, and provide a target and basis for psychological interventions to improve the emotional wellbeing of healthcare workers and enhance their mental health.

## 1 Introduction

Healthcare workers are very valuable resource in the healthcare service industry. In the context of the COVID-19 pandemic, service professionals, including healthcare workers and educators, are often overworked and face significant workloads and psychological pressure (Nucera et al., 2023; Chirico et al., 2022). Once stress becomes chronic, it can translate into anxiety, depression, and burnout (Rehman et al., 2023; Chirico et al., 2023). As a high-risk group for psychological distress, medical workers usually experience varying degrees of anxiety, depression, and burnout (Rizzo et al., 2023; Saragih et al., 2021; Sun et al., 2023). The study (Parmar et al., 2022) predicted that occupational psychological distress, such as depression, anxiety, and burnout, are the determinants that affect turnover intention. Therefore, the mental health of healthcare workers needs urgent attention. Although previous studies (Yıldırım and Ashraf, 2024; Golonka et al., 2019; Ryan et al., 2023) have explored the relationship between burnout, anxiety, and depressive symptoms, previous research still only scratches the surface due to the complexity of the relationship between the three, as well as the shortcomings of traditional methods.

The undertaken research aims to use a network approach to investigate the relationship between burnout, anxiety, and depressive symptoms among Chinese healthcare workers. In addition to assessing the stability and accuracy of the observed model, the study identified central and bridging symptoms in the network. Fundamentally, the main motivation for conducting this study is to improve the understanding of occupational psychological distress among Chinese healthcare workers. Considering the complexity of depression, anxiety, and burnout symptoms among healthcare workers, this study constructed a visual network structure to identify core node symptoms and bridge symptoms. The novelty of this study lies in the adoption of an emerging research methodology, network analysis, and a data-driven approach to studying the interactions between variables, which allows him to understand the degree of closeness of the links between variables, which is rapidly emerging and is widely used in the field of psychology due to its visualization of images and its ability to explore the relationships between variables (Wei et al., 2024).

The significance of this study is that it comprehensively analyses the relationship between burnout, anxiety, and depression symptoms, provides a more systematic and macroscopic understanding of the symptom characteristics of the network, compensates for the traditional method of judging the importance of symptoms by the level of scores, and provides a target point for improving the burnout, anxiety, and depression symptoms of healthcare workers to improve the effectiveness of interventions for related symptoms. This study will help hospital management to develop not only policies to reduce anxiety, depression, and burnout in the hospital setting but also strategies to alleviate occupational psychological distress among healthcare workers to prevent employee accidents, which can be harmful to both the employee and the organization. Finally, the study fills a research gap regarding anxiety, depression, and burnout, and its results provide a basis for future research.

The rest of the paper is structured as follows: the second section covers the literature review. The third section contains research methodology presentation. The fourth section elaborates on the results, while sections five and six include the discussions, limitations and areas for future research, respectively. Finally, we present conclusions.

## 2 Literature review

Burnout is defined as an emotionally depleting response of an individual to prolonged work stress and is a psychological syndrome of depersonalization and low achievement (Tavella et al., 2021). Burnout syndrome includes three syndrome characteristics (frenetic, under-challenged, and worn-out) (Farber, 1990). The frenetic includes a kind of people who are highly engaged and dedicated, and their characteristics are that they devote a lot of time and energy to their work (Montero-Marín et al., 2009). The under-challenged is described as an individual who has no interest in work and superficially performs tasks due to a lack of challenge, motivation, or desire to participate (Montero-Marín et al., 2009). The worn-out includes those whose participation in work has decreased to the point of ignoring their job responsibilities (Montero-Marín et al., 2009). Burnout among healthcare workers has become a global public health problem. One meta-analysis (Aymerich et al., 2022) showed that the prevalence of burnout among healthcare workers exposed to COVID-19 was 37%. In another meta-analysis (Ge et al., 2023) of 30 studies covering more than 94 countries showed a global prevalence of nursing burnout of 0.95%, with an increasing trend over time. Several studies have examined the correlates and potential consequences of burnout. A large sample study (Sun et al., 2023) found that access to psychosocial support, health status, relationship with family, and sleep deprivation were common factors for burnout among healthcare workers. The potential consequences of burnout include decreased health status, work performance, job satisfaction, loss of work enthusiasm, and even lead to uncoordinated doctor-patient relationship, poor colleague relationship, absenteeism, and leaving the job, which directly affects the quality and effectiveness of medical services (Beschoner et al., 2019; Hodkinson et al., 2022; Sohrabi et al., 2022; Zhang et al., 2023).

In addition to burnout, anxiety and depression are highly prevalent psychological problems among healthcare workers. A systematic evaluation and meta-analysis (Aymerich et al., 2022) showed that 33% of healthcare workers exposed to COVID-19 experienced depressive symptoms, and 42% experienced anxiety symptoms. Anxiety and depression are also highly prevalent among Chinese healthcare workers. According to a meta-analysis of Chinese healthcare workers (Xiong et al., 2022), the prevalence of moderate to severe anxiety and depression during the pandemic was 17% and 15%, and that of mild to severe anxiety and depression was estimated to be 37 and 39%, respectively. Compared with the general Chinese population, the prevalence of depression and anxiety was high among women and frontline healthcare workers (Bareeqa et al., 2021). A cross-sectional study (Zhang et al., 2021) reported that hardworking and overcommitted public health workers were at higher risk of depression and anxiety. In addition, anxiety and depression have been found to significantly affect the quality of life and productivity of healthcare workers (An et al., 2020; Wong et al., 2024).

Anxiety and depression are highly correlated common psychological disorders and frequently comorbid (Suradom et al., 2020). Anxiety has been shown to be a predictor of depression, preceding depressive episodes (Warner et al., 2008). Combined burnout, anxiety, and depression are also common among healthcare workers. According to two models of burnout, depression is an important determinant of exhaustion, and depression and anxiety also play an important role in explaining burnout syndromes (Golonka et al., 2019). A systematic evaluation and meta-analysis (Koutsimani et al., 2019) showed that symptoms of depression and anxiety were positively associated with high levels of burnout, although these were not overlapping constructs. A mediation effects analysis (Liu et al., 2023) showed that depression acted as a mediator between anxiety and burnout. Another study (Huang et al., 2024) showed that approximately 37.1% of the variance in depression was explained by a component of burnout. A Korean study (Jihn et al., 2021) found that anxiety and depression predicted burnout in hospital health workers.

Network analysis, as a new method for examining the comorbidity of two or more diseases or syndromes, constructs visual symptom network graphs by quantifying complex connections between symptoms. In network analysis, more influential symptoms are placed at the center of the network, and the remaining relevant symptoms are placed around the network, aiming to calculate indicators of the centrality of symptoms in the network (Borsboom and Cramer, 2013). Network analyses are now widely used in the study of psychological conceptualization and psychiatric disorders, and provide a finer granularity of analysis. For example, several studies have used network analysis to elucidate the structure and central symptoms of anxiety and depression (Chen et al., 2024; He et al., 2023; Zainal and Newman, 2024). However, to the best of our knowledge, no studies have examined Chinese healthcare workers with comorbid burnout, depression, and anxiety symptoms via network analysis.

## 3 Research method

### 3.1 Research design and participants

This study used a cross-sectional design with convenience sampling to select physiatrists and nurses with licensed medical practitioners from tertiary-level A medical institutions in Southwest China. We designed an online survey supported by www.wjx.cn, and systematically trained surveyors sent rapid response codes for the survey to physicians and nurses licensed to practice medicine from tertiary-level A medical institutions in Zigong, China. Healthcare professionals who submitted the online questionnaire were considered to have agreed to participate in this study. The Inclusion criteria were: (a) holding a certificate of physician/nurse qualification from the People’s Republic of China; (b) At least six months of clinical or managerial experience (Six months or more of work is required to qualify for clinical work In China.); (c) have basic telephone or computer skills; and (d) Volunteered to participate in this study and sign the informed consent form. Exclusion criteria were: (a) Those who absent from work, vacation or further training; and (b) Those who suffered a major accident or received psychological treatment. The study strictly followed the principles of the Declaration of Helsinki and was approved by the Ethics Committee of the Fourth People’s Hospital of Zigong.

### 3.2 Measurements

#### 3.2.1 The General Information Scale

General data collected include demographic information (gender, age, years of working experience, marital status, whether the child is an only child, health status, occupation, education level, and the presence of children).

#### 3.2.2 Job burnout

Burnout symptoms were assessed using the Chinese version of the BCSQ-36 (Chen et al., 2023). The BCSQ-36 is a burnout measurement tool developed by Montero-Marín and García-Campayo (2010) comprising 36 items divided into three subscales: frenetic, under-challenged, and worn-out. Each subscale follows a 7-point Likert scale, with each entry scored on a scale of 1 to 7, where 1 = completely disagree and 7 = completely agree. Higher scores indicate more severe burnout. The BCSQ-36 showed good reliability and validity in the Chinese cultural context, with Cronbach’s α coefficient was 0.90 (Chen et al., 2023). In this study, Cronbach’s α coefficient of the scale was 0.90.

#### 3.2.3 Depression

Depressive symptoms were assessed using the Chinese version of QIDS-SR16 (Liu et al., 2013). The QIDS-SR16 is a 16-item self-report scale with 9-dimension that assesses residual symptoms and their severity in respondents (Rush et al., 2003). The questionnaire comprises 16 items, which are categorized into four levels ranging from 0 to 3. The total score of the scale ranges from 0 to 27, with higher scores indicating more severe depressive symptoms. The QIDS-SR16 has good psychometric characteristics in the Chinese population. The Cronbach’s α coefficients was 0.82, with good reliability and validity (Liu et al., 2013; Zhen et al., 2020). In this study, Cronbach’s α coefficient of the scale was 0.78.

#### 3.2.4 Anxiety

Anxiety symptoms were assessed using the Chinese version of SAS (Liu et al., 1997). The SAS is a clinical measurement tool developed by Zung (1971) in 1971 to rate patients’ subjective symptoms of anxiety. The SAS comprises 20 items rated on a 4-point Likert scale based on the respondent’s feelings during the last week. The cumulative score for each item is the total SAS score, with a higher total score indicating a greater degree of anxiety. SAS has been widely used in the Chinese population with Cronbach’s α coefficients of 0.85 (Liu et al., 1997; Sun et al., 2012). Cronbach’α for the present sample was 0.670.

### 3.3 Data collection

This cross-sectional study used an anonymous web-based questionnaire to collect information. The purpose and methodology of the study, the questionnaire, and the process of information collection were introduced by the researcher to the head of each hospital. After obtaining the consent of the relevant head of the hospital, the relevant department was formally requested to send the electronic questionnaire to the healthcare workers who met the requirements. The participants were introduced to the study’s purpose and methodology so they could fully understand the questionnaire’s content. All the participants of the study filled informed consent form. The data collection period of this study was from March to April 2023. A total of 846 healthcare professionals completed the survey. After excluding questionnaires that were not answered completely, 826 participants were finally included in the study.

### 3.4 Statistical analysis

We used R software for data statistics and network visualization. A Gaussian graphical model (GGM) was used to fit the data and construct the network (Costantini et al., 2015). In network modeling, each symptom is considered a node, and correlations between individual symptoms are considered edges (Beard et al., 2016). Edges can be positive (depicted by green lines) or negative (depicted by red lines), and the coarseness of the edges reflects the strength of the association between nodes, with thicker edges indicating stronger associations (Fruchterman and Reingold, 1991).

In order to determine the importance of each node in the network and to explore the bridge nodes in the network that act as a bridge between depression, anxiety, and burnout, this study calculated expected influence (EI) and bridge expected influence (BEI) using the bridge function in the qgraph package and the R package network tools (version 1.3.0), respectively (McNally, 2016). The EI was weighted by the absolute magnitude of related edges, and the higher the EI of a node, the more central it is (Blanchard et al., 2021). The BEI value is used to assess the EI of a node from one community on nodes of another community, and nodes with higher bridge expected impacts were thought to potentially have a higher risk of passing on from this symptom cluster to other symptom clusters (McNally, 2016). Bridging nodes are typically the top 20–30% of nodes in the study for bridging strength centrality or expected influence (Jones et al., 2021). Due to the large number of entries in the scale of this study, the points with the top 20% of EI values were used as central nodes, and the nodes with the top 30% of BEI in the network were set as bridging nodes.

Additionally, the stability of the edge weights and the expected impact of the nodes were also assessed using a non-parametric self-help method for calculating the 95% confidence intervals (CIs) and a sample descent self-help method for calculating the correlation stability (CS) coefficients, respectively, which should not be lower than 0.25 and preferably higher than 0.50 (Epskamp et al., 2018). Bootstrap difference tests were used to assess whether the difference between two edge weights or two node expected impacts was statistically significant (α = 0.05).

## 4 Results

### 4.1 Participant characteristics

A total of 826 healthcare workers qualified for this study, including 684 females (82.8%) and 142 males (17.2%). Their mean age was 31.65 ± 7.91 years, and they worked a mean 9.96 ± 8.36 years. Their marital status was 543 married (65.7%), 256 unmarried (31.0%), and 27 divorced (3.3%). 548 were only children (66.3%), and 278 were not (33.7%). 577 were in good health (69.6%), 243 were in fair health (29.4%), and 6 were in poor health (0.7%). 694 participants were nurses (84.0%) and 132 doctors (16.0%). 14 attended secondary school (1.7%), 285 tertiary school (34.5%), 507 bachelor’s degree (61.4%), and 20 master’s degree (2.4%). 508 participants had children (61.5%), and 318 had no children (38.5%). Table 1 presents abbreviations, items, mean scores, EI, and BEI for each node.

**TABLE 1:** Descriptive statistics of the items in the depression–anxiety–burnout network.

Abbreviation		Items	Mean (SD)	EI	BEI
SAS1		More nervous or anxious than usual	1.800 (0.629)	0.588	0.069
SAS2		Scared for no reason	1.410 (0.552)	0.961	0.227
SAS3		Easily upset or frightened	1.490 (0.615)	1.121	0.126
SAS4		Madness	1.200 (0.482)	0.885	0.266
SAS5		Unfortunate premonition	2.250 (1.002)	0.341	0
SAS6		Tremble	1.080 (0.29)	0.669	0.109
SAS7		Body pain	1.520 (0.672)	0.818	0.071
SAS8		Tiredness	1.750 (0.712)	1.116	0.390
SAS9		Akathisia	2.540 (0.980)	0.480	−0.132
SAS10		Tachycardia	1.390 (0.578)	1.031	0.122
SAS11		Dizzy	1.350 (0.561)	1.116	0.096
SAS12		Faintness	1.240 (0.463)	0.921	0.063
SAS13		Breathlessness	1.180 (0.443)	0.778	0.169
SAS14		Tingling in hands and feet	1.110 (0.339)	0.764	0.095
SAS15		Dyspepsia	1.390 (0.613)	0.806	0.185
SAS16		Frequent urination	1.390 (0.608)	0.641	0.189
SAS17		Excessive sweating	2.460 (1.011)	0.501	0.022
SAS18		Facial flushing	1.310 (0.535)	0.587	0.121
SAS19		Sleep disorders	2.470 (0.949)	−0.209	−0.413
SAS20		Have nightmares	1.570 (0.635)	0.735	0.469
QIDS1	Sleep problems	Sleep onset insomnia	2.750 (0.908)	0.332	0.158
		Midnocturnal insomnia			
		Early morning insomnia			
		Hypersomnia			
QIDS2		Sad mood	1.410 (0.611)	0.936	0.546
QIDS3	Appetite, weight change items	Appetite decrease	1.770 (0.894)	0.570	0.204
		Appetite increase			
		Weight decrease			
		Weight increase			
QIDS4		Concentration/decision-making	1.330 (0.545)	0.888	0.272
QIDS5		Outlook (self)	1.260 (0.692)	0.759	0.130
QIDS6		Suicidal ideation	1.110 (0.340)	0.749	0.231
QIDS7		General interest	1.290 (0.628)	0.767	0.142
QIDS8		Energy/Fatigability	1.380 (0.598)	1.190	0.344
QIDS9	Psychomotor slowing/agitation items	Psychomotor slowing	1.310 (0.675)	0.706	0.104
		Psychomotor agitation			
BCSQ1		Frenetic	4.614 (0.788)	0.0241	0.112
BCSQ2		Underchallenged	3.001 (0.928)	0.673	0.397
BCSQ3		Worn-out	3.103 (0.898)	1.010	0.104

### 4.2 Network analysis

**FIGURE 1:**
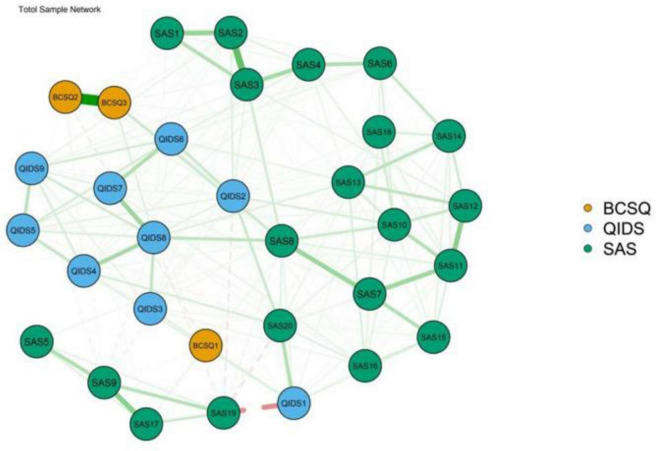
The comorbidity network structure of burnout, depression, and anxiety (*N* = 826).

Figure 1 presents the network visualization of depression, anxiety, and work burnout. The network has 32 nodes, with 237 non-zero edges out of 496 possible edges, and showed excellent stability (0.67 for EI and BEI) and accuracy (Supplementary Figures 1, 2). The most strongly connected edges appeared within their respective communities rather than across communities. Within the depression community, the most significant edge was QIDS7 (General interest)–QIDS8 (Energy/Fatigability) (weight = 0.231). Within the anxiety community, the strongest edge was SAS2 (Scared for no reason)–SAS3 (Easily upset or frightened) (weight = 0.373), followed by SAS9 (Akathisia)–SAS17 (Excessive sweating) (weight = 0.276). Within the burnout community, BCSQ2 (Underchallenged)–BCSQ3 (Worn-out) (weight = 0.613) was the strongest edge. The most robust transdiagnostic edge throughout the community was SAS20 (Have nightmares)–QIDS1 (Sleep problems) (weight = 0.178), followed by SAS8 (Tiredness)–QIDS8 (Energy/Fatigability) (weight = 0.149), SAS20 (Have nightmares)–QIDS2 (Sad mood) (weight = 0.144), and SAS8 (Tiredness)–BCSQ3 (Worn-out) (weight = 0.113). All edge weights are listed in Supplementary Table 1.

**FIGURE 2:**
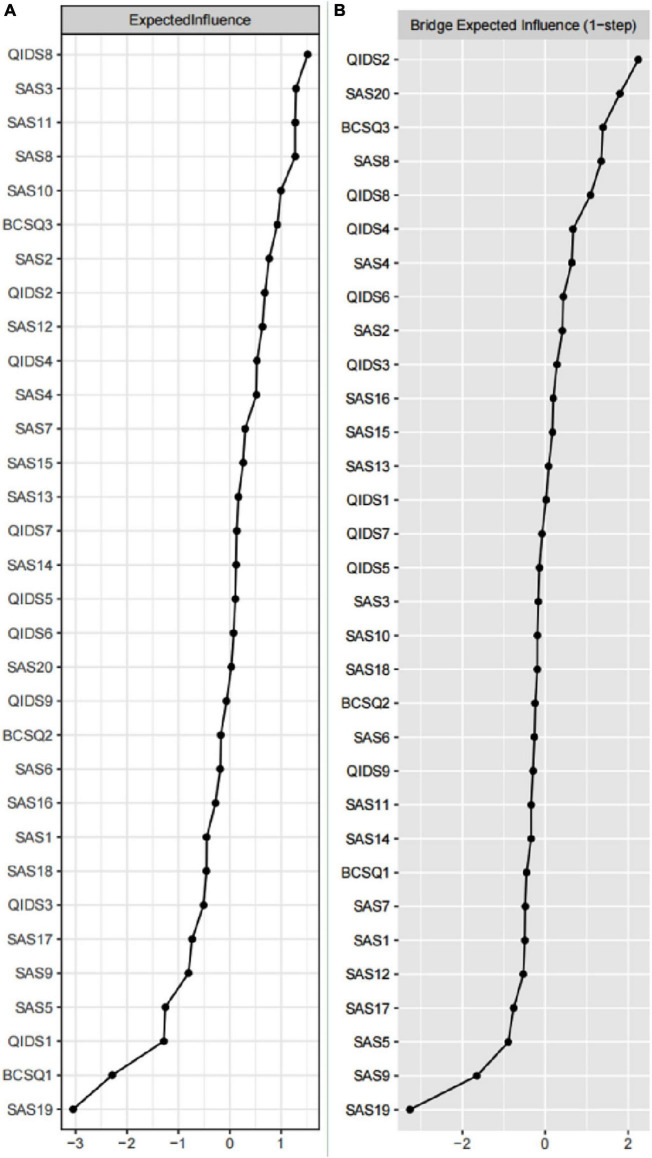
The plot of centrality indices of all symptoms within the network (*N* = 826). The **(A)** is the plot of expected influence, and **(B)** is the plot of bridge expected influence.

**FIGURE 3:**
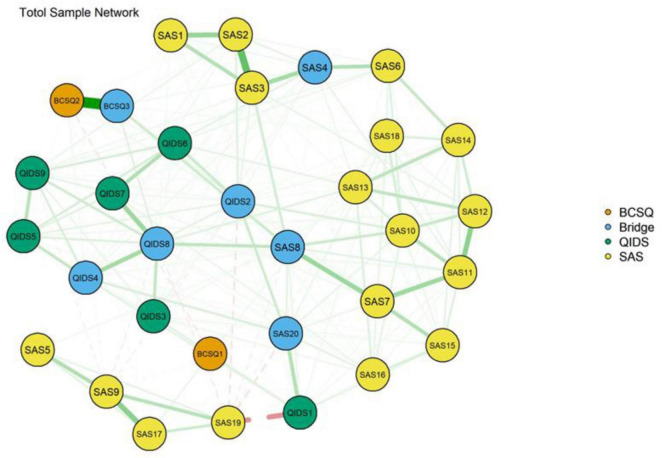
Network structure of burnout, depression, and anxiety showing bridge connections (*N* = 826).

The EI of the nodes is shown in Figure 2A, where the horizontal axis indicates the magnitude of the expected impacts, and the closer to the right side, the larger the expected impacts. As shown in Figure 2A, the nodes with the highest EI are QIDS8 (Energy/Fatigability), SAS3 (Easily upset or frightened), SAS11 (Dizzy), SAS8 (Tiredness), SAS10 (Tachycardia), and BCSQ3 (Worn-out), which suggests that they are strongly and closely linked to other variables in the network. The BEI of the nodes is shown in Figure 2B, where the horizontal axis indicates the magnitude of the expected bridge impact, and the closer to the right side, the larger the expected bridge impact. As shown in Figure 2B, the nodes with the highest BEI are QIDS2 (Sad mood), SAS20 (Have nightmares), BCSQ3 (Worn-out), SAS8 (Tiredness), QIDS8 (Energy/Fatigability), QIDS4 (Concentration/decision-making), and SAS4 (Madness), which suggests that they serve as important bridges within the network. Figure 3 shows the network structure of the burnout, depression, and anxiety cluster with bridging connections, where blue nodes represent bridge nodes, and yellow nodes represent items in the SAS scale. Orange nodes represent items in the BCSQ scale, and green nodes represent items in the QIDS scale. In addition, bootstrapped difference tests between expected influence and bootstrapped difference tests between bridge expected influence are shown in Supplementary Figures 3, 4.

## 5 Discussion

This study explored the complex interactions between the microscopic symptoms of depression, anxiety, and burnout in Chinese healthcare workers using psychological network analysis. We identified several central symptoms [i.e., QIDS8 (Energy/Fatigability), SAS3 (Easily upset or frightened), SAS11 (Dizzy), SAS8 (Tiredness), SAS10 (Tachycardia) and BCSQ3 (Worn-out)] and bridge symptoms [i.e., QIDS2 (Sad mood), SAS20 (Have nightmares), BCSQ3 (Worn-out), SAS8 (Tiredness), QIDS8 (Energy/Fatigability), QIDS4 (Concentration/decision-making) and SAS4 (Madness)]. QIDS8 (Energy/Fatigability), SAS8 (Tiredness), and BCSQ3 (Worn-out) were both central and bridge symptoms.

Our findings suggest that there may be comorbidities of burnout, depression, and anxiety, which is consistent with previous studies involving medical students (Ernst et al., 2021; Peng et al., 2023). However, burnout is conceptually distinct from anxiety or depression, and burnout has been categorized previously as a separate work-related phenomenon (de Amorim Macedo et al., 2023). The core criteria of anxiety and depression may not apply to burnout, as burnout may focus only on anxiety and worry associated with occupational stress. Depression is more closely related to burnout than anxiety, possibly because burnout and depression are similarly related to work stressors (Verkuilen et al., 2021). Burnout is a state of physical and mental fatigue and exhaustion that occurs when an individual is under the weight of work, and depression can also be caused by job stress. The complex and unique relationship between burnout, depression, and anxiety provides further evidence that symptoms of burnout in healthcare workers should not be viewed as a single, unified syndrome, but rather as a multidimensional syndrome, consistent with previous research (Bianchi et al., 2015). Moreover, our results suggest that burnout, anxiety, and depression are closely related on one hand, but, on the other, each has its unique characteristics. This finding provides a plausible explanation for why the debate of whether burnout overlaps with depression or anxiety continues to this day (Ahola et al., 2005; Bianchi et al., 2015).

The present study shows that depression and anxiety symptoms seem to present a closer link in the symptom network. The co-morbid relationship between the two seems to exist not only in the specific group of healthcare workers. With regard to the co-morbidity between the two, the US National Comorbidity Survey stated that approximately 50% of people with major depressive disorder have one or more anxiety disorders. Anxiety symptoms and depressive symptoms presented by different populations have been shown to be highly correlated (Beard et al., 2016; Kessler et al., 2003; Park and Kim, 2020; Rouquette et al., 2018). The interdependence between depression and anxiety has long been proposed and described, with early conceptualizations of depression emphasizing the central role of anxiety in depression (Bui and Fava, 2017). The American Psychological Association stated that the depressive response is considered an attenuated form of anxiety (Mendelson, 1995). There are multiple possible mechanisms for anxiety-depression co-morbidity and symptom correlation, one of which is that the two share a common pathogenic mechanism. Current hypotheses to explain this common pathogenic mechanism include dysfunction in the serotonergic system (Bui and Fava, 2017), and altered activation and connectivity of the ventral cingulate gyrus and amygdala (Niu et al., 2017).

Previous studies have found that, in networks composed of different communities, the strongest edges generally exist within communities rather than cross-community edges (Li et al., 2023). The results of the network analysis in this study reaffirmed this finding. Specifically, the strongest edges were present in the burnout community [i.e., BCSQ2 (Underchallenged)–BCSQ3 (Worn-out)], anxiety community [i.e., SAS2 (Scared for no reason)–SAS3 (Easily upset or frightened) and SAS9 (Akathisia)–SAS17 (Excessive sweating)], and depression community [i.e., QIDS7 (General interest)–QIDS8 (Energy/Fatigability)]. The close relationship between BCSQ2 (Underchallenged)–BCSQ3 (Worn-out) has been supported by previous studies. The under-challenged subtype describes that individuals respond to work superficially through indifference and detachment. Although they have not been ignored, they have not participated much. The worn-out subtype describes that individuals cope with work-related difficulties by neglecting their responsibilities, that is, they lack participation in their work so that they will give up when facing any difficulties (Montero-Marín et al., 2009). In this study, the relationship between them is the closest, which may be because theoretically, the three subtypes of job burnout can be divided into a continuum according to their devotion to work-related tasks, from frenetic subtype (high devotion) to under-challenged subtype (moderate devotion) to worn-out subtype (low devotion), which correspond to the values of participation, indifference and neglect. Of these, BCSQ2 (Underchallenged) and BCSQ3 (Worn-out) reflect the deterioration of burnout symptoms from moderate to severe (Montero-Marín and García-Campayo, 2010). In addition, SAS2 (Scared for no reason)–SAS3 (Easily upset or frightened) and SAS9 (Akathisia)–SAS17 (Excessive sweating) are the edges connecting the second and third strongest in the network, respectively. “Scared for no reason” refers to a lesser degree of fear, which is often an internal experience, while “Easily upset or frightened” refers to a heavier degree of intense and uncontrollable fear, which is often accompanied by physical reaction, that is, the long-term accumulation of fear leads to the expression of strong panic (Craske et al., 2017). Previous studies (Zyss et al., 2009) have emphasized the close relationship between akathisia and hyperhidrosis. Akathisia is a kind of restless movement, which is manifested by uncontrollable agitation, body restlessness, and the uncomfortable feeling that he can’t sit still because he wants to walk back and forth or stand still. Subjectively, patients have the impulse to be restless and forced to move, and objectively, they often perform repetitive movements, especially in the lower limbs, such as constantly swinging or crossing their legs, constantly exchanging the relative positions of their legs, constantly pacing and fidgeting, resulting in Excessive sweating. The possible explanation for the strong connection between QIDS7 (General interest) and QIDS8 (Energy/Fatigability) is that the huge workload and pressure make medical workers devote most of their energy to their work, unable to balance the time between work and pursuing interests and hobbies, and the interest level decreases over time.

Furthermore, strong cross-community edges are key edges that lead to activation and contagion between different communities, which is essential to the emergence and persistence of symbiotic psychological issues. Specifically, this study identified four strong cross-community edges: SAS20 (Have nightmares)–QIDS1 (Sleep problems), SAS8 (Tiredness)–QIDS8 (Energy/Fatigability), SAS20 (Have nightmares)–QIDS2 (Sad mood) and SAS8 (Tiredness)–BCSQ3 (Worn-out). These findings hint the critical pathways of SAS20 (Have nightmares)–QIDS1 (Sleep problems), SAS8 (Tiredness)–QIDS8 (Energy/Fatigability), SAS20 (Have nightmares)–QIDS2 (Sad mood) may be the underlying mechanisms for the strong association between anxiety and depression, often leading to their co-occurrence. Sleep disorder mainly refers to abnormal sleep volume; Abnormal behaviors including night terrors and nightmares (Akman et al., 2015) often occur during sleep and are often accompanied by anxiety, depression, and other negative emotions (Yennurajalingam et al., 2016). With regard to the connection between SAS20 (Have nightmares) and QIDS1 (Sleep problems), the possible explanation is that nightmares can lead to insomnia, and people cannot sleep because they are afraid of nightmares (Stefani and Högl, 2021). As for the connection between SAS8 (Tiredness) and QIDS8 (Energy/Fatigability), the possible reason is that medical workers can’t stay energetic due to physical and psychological fatigue, and then feel weak. About the connection between SAS20 (Have nightmares) and QIDS2 (Sad mood), it may be that frequent nightmares cause sadness. Additionally, the connective pathway between SAS8 (Tiredness) and BCSQ3 (Worn-out) may be the key connection that activates the development of anxiety and burnout. One of the characteristics of the worn-out subtype is “lack of control,” describing feelings of powerlessness due to dealing with uncontrollable situations (Montero-Marín and García-Campayo, 2010), which may go some way to explaining the relationship between fatigue and exhaustion in healthcare workers. Previous research has tended to support the conclusion that depression, anxiety, and burnout co-exist and that the four strong trans-community margins described above may be a key underlying mechanism that leads to the co-existence of all three, which needs to be explored in further research.

Centrality results indicated that QIDS8 (Energy/Fatigability) had the highest EI, followed by SAS3 (Easily upset or frightened), SAS11 (Dizzy), SAS8 (Tiredness), SAS10 (Tachycardia), and BCSQ3 (Worn-out). In other words, QIDS8 (Energy/Fatigability) was the most central symptom in the anxiety-depression-burnout network of healthcare workers and should be considered the driver and trigger of all psychological stress symptoms. A previous study on COPD patients (Yohannes et al., 2022) also found that “Energy” had a high centrality weight and was one of the central symptoms activating a network of depression and anxiety symptoms. Another study on nasopharyngeal cancer patients (Zhan et al., 2024) also confirmed the strong centrality of “Energy.” According to the DSM-5, low energy is a major symptom of depression, while panic, dizziness, fatigue, and palpitations are possible physical signs of anxiety (American Psychiatric Association, DSMTF and Association, American Psychiatric, 2013). We speculate that the lack of energy in healthcare workers makes it difficult for them to remain enthusiastic and focused on their work, which in turn trigger depression, anxiety, and burnout. Additionally, the EI for SAS3 (Easily upset or frightened) was second only to that for QIDS8 (Energy/Fatigability), suggesting that “Easily upset or frightened” may be another important trigger in the network with a high potential to activate other neighboring symptoms and further lead to serious mental problems. The centrality of “Easily upset or frightened” is confirmed by the above results on the edges, that is, SAS2 (Scared for no reason)–SAS3 (Easily upset or frightened) has the second strongest connection in the entire network. Fright is a particularly strong fear conditioning that can lead to excessive psychophysiological responses to stimuli (Olivera-Pasilio and Dabrowska, 2023) and may therefore feature prominently in the depression-anxiety-burnout network. This study highlights the urgency of prioritizing screening and intervention for QIDS8 (Energy/Fatigability), SAS3 (Easily upset or frightened), SAS11 (Dizzy), SAS8 (Tiredness), SAS10 (Tachycardia), and BCSQ3 (Worn-out) symptoms in healthcare workers to minimize the impact on mental health.

The results of bridge centrality suggested that QIDS2 (Sad mood), SAS20 (Have nightmares), BCSQ3 (Worn-out), SAS8 (Tiredness), QIDS8 (Energy/Fatigability), QIDS4 (Concentration/decision-making) and SAS4 (Madness) had the highest BEI and should be considered key bridge symptoms leading to the comorbidity of depression, anxiety and burnout. The top three were QIDS2 (Sad mood), SAS20 (Have nightmares), and BCSQ3 (Worn-out). QIDS2 (Sad mood) was the most critical bridge symptom in the network, a finding supported by a large-sample systematic evaluation (Cai et al., 2024). This systematic evaluation identified sadness as the most common bridge-centered symptom in the depression-anxiety network. BCSQ3 (Worn-out) was also an important bridge symptom, which is consistent with the results of a study (Peng et al., 2023) of the network of burnout, depression, anxiety, and dropout intention in undergraduate medical students. In addition, the above marginal results suggest that the bridging of these nodes may be realized through specific pathways, e.g., the bridging of SAS8 (Tiredness) may be realized through the SAS8 (Tiredness)–QIDS8 (Energy/Fatigability) energy level, and SAS8 (Tiredness)–BCSQ3 (Worn-out) specific pathways. This finding provides insights for hospital administrators looking to target interventions that are less labor intensive and more effective, i.e., interventions that target “Tiredness” may achieve a more significant double or even triple-stacking effect. Specifically, when healthcare workers are severely anxious, interventions targeting “SAS8 (Tiredness)” not only help to alleviate anxiety but also reduce the psychiatric crises of depression and burnout through an intrinsic link to QIDS8 (Energy/Fatigability) and BCSQ3 (Worn-out). It is also worth noting that SAS8 (Tiredness), BCSQ3 (Worn-out), and QIDS8 (Energy/Fatigability) are important central as well as bridging symptoms in the current network model. These findings suggest that interventions aimed at reducing SAS8 (Tiredness), BCSQ3 (Worn-out), and QIDS8 (Energy/Fatigability) in Southwest China, may not only reduce anxiety-depressive symptoms but also reduce the incidence of burnout.

## 6 Implications

This study has several implications for clinical practice and management. In order to detect and reduce depression, anxiety and burnout among healthcare workers early, hospital administrators and policymakers must conduct timely screening and targeted interventions for specific symptoms. Specifically, to address the psychological symptoms of “Energy/Fatigability,” managers can take necessary interventions, including mindfulness-based interventions, cognitive behavioral therapy, stress inoculation therapy, psychoeducation and stress management (Moore et al., 2024). For the “Easily upset or frightened” symptoms, Managers can organize startle-relieving workshops and invite healthcare workers to share measures to mitigate the startle response to help healthcare workers develop optimal coping strategies (Luger and Koziol, 2024). For the “Have nightmares,” moderate-intensity aerobic exercise, which includes walking at a moderate pace, water aerobics, dancing, recreational swimming, gardening, table tennis, and climbing stairs at a moderate pace, is a cost-effective health-promoting strategy that improves the quality and duration of sleep (Okechukwu et al., 2022). Nursing managers must be vigilant about “Sad mood” in the depressed population and should implement potential prevention strategies as soon as possible. Healthcare organizations can conduct online surveys for early screening of depressed nurses and provide timely professional psychotherapy (Moore et al., 2024). For the “Worn-out” symptoms in the burnout group, managers can organize appropriate relaxation training for healthcare workers before their shifts, such as positive thinking meditation, breathing relaxation or playing some soothing music (Jones, 2019). This can improve their mental health as well as their professional performance.

## 7 Limitations

Our study has several limitations. Firstly, due to the cross-sectional nature of the study design, we were unable to determine causal relationships or long-term dynamic changes between symptoms, although the primary findings provide a basis for future highly focused causal hypotheses. Future studies should conduct longitudinal cross-lagged network analyses to determine the direction between psychiatric symptoms and provide more accurate information for interventions. Second, the use of convenience sampling may affect the representativeness of our sample. The majority of subjects were female, which may affect the generalizability of the results. Future studies should include more male healthcare workers or verify the applicability of our results in other populations. Thirdly, the assessments used in our study were based on self-reports and therefore may be subject to recall bias and/or social desirability bias. Fourth, symptoms of depression, anxiety, and burnout were highly correlated with factors such as social support, workload, and economic conditions, which we did not collect in this study. Future research needs to further examine how these factors affect the depression-anxiety-burnout network in the healthcare worker population, thus providing valuable insights into the prevention and treatment of depression, anxiety and burnout in healthcare workers. Finally, as this study only recruited healthcare workers in Southwest China, caution should be exercised in generalizing the findings to populations in other countries. There is a need to extend the study to healthcare workers in other countries around the world to further assess the external validity of the findings.

## 8 Conclusion

In summary, our study assessed the network structure of depression, anxiety, and symptoms among healthcare workers in Southwest China. We found that QIDS8 (Energy/Fatigability) was the strongest central symptom in the network, whereas QIDS2 (Sad mood), SAS20 (Have nightmares), BCSQ3 (Worn-out), SAS8 (Tiredness), QIDS8 (Energy/Fatigability), QIDS4 (Concentration/decision-making) and SAS4 (Madness) were the bridging symptoms in the network. These findings provide new insights into the symptom-symptom relationships of depression, anxiety, and burnout, and are valuable for the prevention and treatment of these three common psychiatric disorders among healthcare workers.

## Data Availability

The raw data supporting the conclusions of this article will be made available by the authors, without undue reservation.

## Supplementary material

Supplementary Figure 1 Centrality stability for network.

Supplementary Figure 2 Edge-weight accuracy for network.

Supplementary Figure 3 Bootstrapped difference tests between expected influence.

Supplementary Figure 4 Bootstrapped difference tests between bridge expected influence.

Supplementary Table 1 Edge weights between all nodes of network.

## References

[B1] Ahola K. Honkonen T. Isometsä E. Kalimo R. Nykyri E. Aromaa A. (2005). The relationship between job-related burnout and depressive disorders–results from the Finnish Health 2000 Study. *J. Affect. Disord.* 88 55–62. 10.1016/j.jad.2005.06.004 16038984

[B2] Akman T. Yavuzsen T. Sevgen Z. Ellidokuz H. Yilmaz A. U. (2015). Evaluation of sleep disorders in cancer patients based on Pittsburgh Sleep Quality Index. *Eur. J. Cancer Care* 24 553–559. 10.1111/ecc.12296 25727241

[B3] American Psychiatric Association, DSMTF and Association, American Psychiatric (2013). *Diagnostic and statistical manual of mental disorders: DSM-5*. Washington, DC: American Psychiatric Association.

[B4] An Y. Yang Y. Wang A. Li Y. Zhang Q. Cheung T. (2020). Prevalence of depression and its impact on quality of life among frontline nurses in emergency departments during the COVID-19 outbreak. *J. Affect. Disord.* 276 312–315. 10.1016/j.jad.2020.06.047 32871661 PMC7361044

[B5] Aymerich C. Pedruzo B. Pérez J. L. Laborda M. Herrero J. Blanco J. (2022). COVID-19 pandemic effects on health worker’s mental health: systematic review and meta-analysis. *Eur. Psychiatry* 65:e10. 10.1192/j.eurpsy.2022.1 35060458 PMC8828390

[B6] Bareeqa S. B. Ahmed S. I. Samar S. S. Yasin W. Zehra S. Monese G. M. (2021). Prevalence of depression, anxiety and stress in china during COVID-19 pandemic: a systematic review with meta-analysis. *Int. J. Psychiatry Med.* 56 210–227. 10.1177/0091217420978005 33243029

[B7] Beard C. Millner A. J. Forgeard M. J. Fried E. I. Hsu K. J. Treadway M. T. (2016). Network analysis of depression and anxiety symptom relationships in a psychiatric sample. *Psychol. Med.* 46 3359–3369. 10.1017/S0033291716002300 27623748 PMC5430082

[B8] Beschoner P. Limbrecht-Ecklundt K. Jerg-Bretzke L. (2019). Mental health among physicians: burnout, depression, anxiety and substance abuse in the occupational context. *Der Nervenarzt* 90 961–974. 10.1007/s00115-019-0739-x 31172233

[B9] Bianchi R. Schonfeld I. S. Laurent E. (2015). Burnout-depression overlap: a review. *Clin. Psychol. Rev.* 36 28–41. 10.1016/j.cpr.2015.01.004 25638755

[B10] Blanchard M. A. Roskam I. Mikolajczak M. Heeren A. (2021). A network approach to parental burnout. *Child Abuse Neglect* 111:104826. 10.1016/j.chiabu.2020.104826 33310372

[B11] Borsboom D. Cramer A. O. (2013). Network analysis: an integrative approach to the structure of psychopathology. *Annu. Rev. Clin. Psychol.* 9 91–121. 10.1146/annurev-clinpsy-050212-185608 23537483

[B12] Bui E. Fava M. (2017). From depression to anxiety, and back. *Acta Psychiatr. Scand.* 136 341–342. 10.1111/acps.12801 28865404

[B13] Cai H. Chen M. Y. Li X. H. Zhang L. Su Z. Cheung T. (2024). A network model of depressive and anxiety symptoms: a statistical evaluation. *Mol. Psychiatry* 29 767–781. 10.1038/s41380-023-02369-5 38238548 PMC11153039

[B14] Chen S. Z. Zainal N. H. Newman M. G. (2024). Elevated depression and anxiety predict future patterns of individualistic and collectivistic cultural values: a cross-lagged longitudinal network analysis. *J. Affect. Disord.* 349 310–320. 10.1016/j.jad.2023.12.083 38181844 PMC10950001

[B15] Chen Y. Nie C. Bu W. You W. Xu P. (2023). Reliability and validity of the Chinese version of the burnout clinical subtypes questionnaire. *Contempor. Nurse* 30 11–17.

[B16] Chirico F. Batra K. Batra R. Ferrari G. Crescenzo P. Nucera G. (2023). Spiritual well-being and burnout syndrome in healthcare: a systematic review. *J. Health Soc. Sci.* 8:13.

[B17] Chirico F. Crescenzo P. Nowrouzi-Kia B. Tarchi L. Batra K. Ferrari G. (2022). Prevalence and predictors of burnout syndrome among schoolteachers during the COVID-19 pandemic in Italy: a cross-sectional survey. *J. Health Soc. Sci.* 7 195–211. 10.19204/2022/PRVL6

[B18] Costantini G. Epskamp S. Borsboom D. Perugini M. Mõttus R. Waldorp L. J. (2015). State of the aRt personality research: a tutorial on network analysis of personality data in R. *J. Res. Pers.* 54 13–29.

[B19] Craske M. G. Stein M. B. Eley T. C. Milad M. R. Holmes A. Rapee R. M. (2017). Anxiety disorders. *Nat. Rev. Dis. Prim.* 3:17024. 10.1038/nrdp.2017.24 28470168 PMC11009418

[B20] de Amorim Macedo M. J. de Freitas C. P. P. Bermudez M. B. Souza Vazquez A. C. Salum G. A. Dreher C. B. (2023). The shared and dissociable aspects of burnout, depression, anxiety, and irritability in health professionals during COVID-19 pandemic: a latent and network analysis. *J. Psychiatr. Res.* 166 40–48. 10.1016/j.jpsychires.2023.09.005 37738779

[B21] Epskamp S. Borsboom D. Fried E. I. (2018). Estimating psychological networks and their accuracy: a tutorial paper. *Behav. Res. Methods* 50 195–212. 10.3758/s13428-017-0862-1 28342071 PMC5809547

[B22] Ernst J. Jordan K. D. Weilenmann S. Sazpinar O. Gehrke S. Paolercio F. (2021). Burnout, depression and anxiety among Swiss medical students–A network analysis. *J. Psychiatr. Res.* 143 196–201. 10.1016/j.jpsychires.2021.09.017 34500349

[B23] Farber B. A. (1990). Burnout in psychotherapists: incidence, types, and trends. *Psychother. Private Pract.* 8 35–44.

[B24] Fruchterman T. M. Reingold E. M. (1991). Graph drawing by force-directed placement. *Softw. Pract. Exp.* 21 1129–1164.

[B25] Ge M. W. Hu F. H. Jia Y. J. Tang W. Zhang W. Q. Chen H. L. (2023). Global prevalence of nursing burnout syndrome and temporal trends for the last 10 years: a meta-analysis of 94 studies covering over 30 countries. *J. Clin. Nurs.* 32 5836–5854. 10.1111/jocn.16708 37194138

[B26] Golonka K. Mojsa-Kaja J. Blukacz M. Gawłowska M. Marek T. (2019). Occupational burnout and its overlapping effect with depression and anxiety. *Int. J. Occup. Med. Environ. Health* 32 229–244. 10.13075/ijomeh.1896.01323 30855601

[B27] He Y. Wu C. LeMoult J. Huang J. Zhao Y. Liang K. (2023). Exploring symptom-level associations between anxiety and depression across developmental stages of adolescence: a network analysis approach. *BMC Psychiatry* 23:941. 10.1186/s12888-023-05449-6 38093232 PMC10720222

[B28] Hodkinson A. Zhou A. Johnson J. Geraghty K. Riley R. Zhou A. (2022). Associations of physician burnout with career engagement and quality of patient care: systematic review and meta-analysis. *BMJ* 378:e070442. 10.1136/bmj-2022-070442 36104064 PMC9472104

[B29] Huang C. P. Zou J. M. Ma H. Zhong Y. (2024). Role stress, occupational burnout and depression among emergency nurses: a cross-sectional study. *Int. Emerg. Nurs.* 72:101387. 10.1016/j.ienj.2023.101387 37984024

[B30] Jihn C. H. Kim B. Kim K. S. (2021). Predictors of burnout in hospital health workers during the COVID-19 outbreak in South Korea. *Int. J. Environ. Res. Public Health* 18:11720. 10.3390/ijerph182111720 34770231 PMC8582777

[B31] Jones B. (2019). Fifteen minutes may decrease nursing burnout: a discussion paper. *Int. J. Nurs. Sci.* 7 121–123. 10.1016/j.ijnss.2019.11.004 32099870 PMC7031112

[B32] Jones P. J. Ma R. McNally R. J. (2021). Bridge centrality: a network approach to understanding comorbidity. *Multivar. Behav. Res.* 56 353–367. 10.1080/00273171.2019.1614898 31179765

[B33] Kessler R. C. Berglund P. Demler O. Jin R. Koretz D. Merikangas K. R. (2003). The epidemiology of major depressive disorder: results from the National Comorbidity Survey Replication (NCS-R). *JAMA* 289 3095–3105. 10.1001/jama.289.23.3095 12813115

[B34] Koutsimani P. Montgomery A. Georganta K. (2019). The relationship between burnout, depression, and anxiety: a systematic review and meta-analysis. *Front. Psychol.* 10:284. 10.3389/fpsyg.2019.00284 30918490 PMC6424886

[B35] Li Y. Guo Z. Tian W. Wang X. Dou W. Chen Y. (2023). An investigation of the relationships between suicidal ideation, psychache, and meaning in life using network analysis. *BMC Psychiatry* 23:257. 10.1186/s12888-023-04700-4 37069569 PMC10111716

[B36] Liu F. Zhao Y. Chen Y. Tu Z. (2023). The mediation effect analysis of nurse’s mental health status and burnout under COVID-19 epidemic. *Front. Public Health* 11:1221501. 10.3389/fpubh.2023.1221501 37915821 PMC10616456

[B37] Liu J. Xiang Y. T. Wang G. Zhu X. Z. Ungvari G. S. Kilbourne A. M. (2013). Psychometric properties of the Chinese versions of the Quick Inventory of Depressive Symptomatology - Clinician Rating (C-QIDS-C) and Self-Report (C-QIDS-SR). *J. Affect. Disord.* 147 421–424. 10.1016/j.jad.2012.08.035 22995944

[B38] Liu X. C. Oda S. Peng X. Asai K. (1997). Life events and anxiety in Chinese medical students. *Soc. Psychiatry Psychiatr. Epidemiol.* 32 63–67. 10.1007/BF00788922 9050346

[B39] Luger S. Koziol D. (2024). Authentic nursing leadership theory and nurse leaders’ stories: storytelling workshop impact on nurse leader burnout. *Nurs. Manag.* 55 22–27. 10.1097/nmg.0000000000000129 38690861

[B40] McNally R. J. (2016). Can network analysis transform psychopathology? *Behav. Res. Ther.* 86 95–104. 10.1016/j.brat.2016.06.006 27424882

[B41] Mendelson G. (1995). *Diagnostic and Statistical Manual of Mental Disorders, (DSM-IV).* Washington, DC: American Psychiatric Association.

[B42] Montero-Marín J. García-Campayo J. (2010). A newer and broader definition of burnout: validation of the “Burnout Clinical Subtype Questionnaire (BCSQ-36)”. *BMC Public Health* 10:302. 10.1186/1471-2458-10-302 20525178 PMC2887826

[B43] Montero-Marín J. García-Campayo J. Mosquera Mera D. López del Hoyo Y. (2009). A new definition of burnout syndrome based on Farber’s proposal. *J. Occup. Med. Toxicol. (London, England)* 4:31. 10.1186/1745-6673-4-31 19948055 PMC2794272

[B44] Moore C. Kelly S. Melnyk B. M. (2024). The use of mHealth apps to improve hospital nurses’ mental health and well-being: a systematic review. *Worldviews Evid. Based Nurs.* 21 110–119. 10.1111/wvn.12716 38491775

[B45] Niu M. Wang Y. Jia Y. Wang J. Zhong S. Lin J. (2017). Common and specific abnormalities in cortical thickness in patients with major depressive and bipolar disorders. *Ebiomedicine* 16 162–171. 10.1016/j.ebiom.2017.01.010 28109831 PMC5474436

[B46] Nucera G. Chirico F. Yildirim M. Szarpak L. Magnavita N. (2023). Addressing burnout and PTSD among paramedics and emergency staff after the COVID-19 pandemic: the role of occupational health services and workplace health promotion programs. *Disaster Emerg. Med. J.* 8 131–133.

[B47] Okechukwu C. E. Masala D. D’Ettorre G. La Torre G. (2022). Moderate-intensity aerobic exercise as an adjunct intervention to improve sleep quality among rotating shift nurses. *Clin. Ter.* 173 184–186. 10.7417/CT.2022.2414 35385043

[B48] Olivera-Pasilio V. Dabrowska J. (2023). Fear-conditioning to unpredictable threats reveals sex and strain differences in rat Fear-Potentiated Startle (FPS). *Neuroscience* 530 108–132. 10.1016/j.neuroscience.2023.08.030 37640137 PMC10726736

[B49] Park S. C. Kim D. (2020). The centrality of depression and anxiety symptoms in major depressive disorder determined using a network analysis. *J. Affect. Disord.* 271 19–26. 10.1016/j.jad.2020.03.078 32312693

[B50] Parmar V. Channar Z. A. Ahmed R. R. Streimikiene D. Pahi M. H. Streimikis J. (2022). Assessing the organizational commitment, subjective vitality and burnout effects on turnover intention in private universities. *Oeconomia Copernicana* 13 251–286.

[B51] Peng P. Chen S. Hao Y. He L. Wang Q. Zhou Y. (2023). Network of burnout, depression, anxiety, and dropout intention in medical undergraduates. *Int. J. Soc. Psychiatry* 69 1520–1531. 10.1177/00207640231166629 37092762

[B52] Rehman U. Yıldırım M. Shahnawaz M. G. (2023). A longitudinal study of depression, anxiety, and stress among Indians during COVID-19 pandemic. *Psychol. Health Med.* 28 60–68. 10.1080/13548506.2021.2023751 34974787

[B53] Rizzo A. Yıldırım M. Öztekin G. G. Carlo A. D. Nucera G. Szarpak Ł (2023). Nurse burnout before and during the COVID-19 pandemic: a systematic comparative review. *Front. Public Health* 11:1225431. 10.3389/fpubh.2023.1225431 37732086 PMC10507882

[B54] Rouquette A. Pingault J. B. Fried E. I Orri M. Falissard B. Kossakowski J. J. (2018). Emotional and behavioral symptom network structure in elementary school girls and association with anxiety disorders and depression in adolescence and early adulthood: a network analysis. *JAMA Psychiatry* 75 1173–1181. 10.1001/jamapsychiatry.2018.2119 30128480 PMC6248096

[B55] Rush A. J. Trivedi M. H. Ibrahim H. M. Carmody T. J. Arnow B. Klein D. N. (2003). The 16-item Quick Inventory of Depressive Symptomatology (QIDS), clinician rating (QIDS-C), and self-report (QIDS-SR): a psychometric evaluation in patients with chronic major depression. *Biol. Psychiatry* 54 573–583. 10.1016/s0006-3223(02)01866-8 12946886

[B56] Ryan E. Hore K. Power J. Jackson T. (2023). The relationship between physician burnout and depression, anxiety, suicidality and substance abuse: a mixed methods systematic review. *Front. Public Health* 11:1133484. 10.3389/fpubh.2023.1133484 37064688 PMC10098100

[B57] Saragih I. D. Tonapa S. I. Saragih I. S. Advani S. Batubara S. O. Suarilah I. (2021). Global prevalence of mental health problems among healthcare workers during the COVID-19 pandemic: a systematic review and meta-analysis. *Int. J. Nurs. Stud.* 121:104002. 10.1016/j.ijnurstu.2021.104002 34271460 PMC9701545

[B58] Sohrabi Y. Yarmohammadi H. Pouya A. B. Arefi M. F. Hassanipour S. Poursadeqiyan M. (2022). Prevalence of job burnout in Iranian nurses: a systematic review and meta-analysis. *Work* 73 937–943. 10.3233/WOR-210283 35988237

[B59] Stefani A. Högl B. (2021). Nightmare disorder and isolated sleep paralysis. *Neurotherapeutics* 18 100–106. 10.1007/s13311-020-00966-8 33230689 PMC8116464

[B60] Sun H. Zhang T. Wang X. Wang C. Zhang M. Song H. (2023). The occupational burnout among medical staff with high workloads after the COVID-19 and its association with anxiety and depression. *Front. Public Health* 11:1270634. 10.3389/fpubh.2023.1270634 37954047 PMC10639132

[B61] Sun W. Fu J. Chang Y. Wang L. (2012). Epidemiological study on risk factors for anxiety disorder among Chinese doctors. *J. Occupat. Health* 54 1–8. 10.1539/joh.11-0169-oa 22156318

[B62] Suradom C. Wongpakaran N. Wongpakaran T. Lerttrakarnnon P. Jiraniramai S. Taemeeyapradit U. (2020). Mediation model of comorbid anxiety disorders in late-life depression. *Ann. Gen. Psychiatry* 19:63. 10.1186/s12991-020-00313-3 33292322 PMC7670777

[B63] Tavella G. Hadzi-Pavlovic D. Parker G. (2021). Burnout: redefining its key symptoms. *Psychiatry Res.* 302:114023. 10.1016/j.psychres.2021.114023 34052460

[B64] Verkuilen J. Bianchi R. Schonfeld I. S. Laurent E. (2021). Burnout-depression overlap: exploratory structural equation modeling bifactor analysis and network analysis. *Assessment* 28 1583–1600. 10.1177/1073191120911095 32153199

[B65] Warner V. Wickramaratne P. Weissman M. M. (2008). The role of fear and anxiety in the familial risk for major depression: a three-generation study. *Psychol. Med.* 38 1543–1556. 10.1017/S0033291708002894 18275630 PMC2904071

[B66] Wei H. Liu M. Wang Z. Qu W. Zhang S. Zhang B. (2024). Anxiety, depression, and post-traumatic stress disorder in nurses exposed to horizontal violence: a network analysis. *BMC Nurs.* 23:750. 10.1186/s12912-024-02408-8 39396956 PMC11472536

[B67] Wong K. W. Wu X. Dong Y. (2024). Interventions to reduce burnout and improve the mental health of nurses during the COVID-19 pandemic: a systematic review of randomised controlled trials with meta-analysis. *Int. J. Ment. Health Nurs.* 33 324–343. 10.1111/inm.13251 37985559

[B68] Xiong N. Fritzsche K. Pan Y. Löhlein J. Leonhart R. (2022). The psychological impact of COVID-19 on Chinese healthcare workers: a systematic review and meta-analysis. *Soc. Psychiatry Psychiatr. Epidemiol.* 57 1515–1529. 10.1007/s00127-022-02264-4 35325261 PMC8943357

[B69] Yennurajalingam S. Tayjasanant S. Balachandran D. Padhye N. S. Williams J. L. Liu D. D. (2016). Association between daytime activity, fatigue, sleep, anxiety, depression, and symptom burden in advanced cancer patients: a preliminary report. *J. Palliat. Med.* 19 849–856. 10.1089/jpm.2015.0276 27148765 PMC4982947

[B70] Yıldırım M. Ashraf F. (2024). Fear of COVID-19, coronavirus anxiety, COVID-19 burnout, and resilience: examining psychometric properties of COVID-19 burnout scale in Urdu. *J. Asian Afric. Stud.* 59 2303–2315.

[B71] Yohannes A. M. Murri M. B. Hanania N. A. Regan E. A. Iyer A. Bhatt S. P. (2022). Depressive and anxiety symptoms in patients with COPD: a network analysis. *Respir. Med.* 198:106865. 10.1016/j.rmed.2022.106865 PMC10698756 35576775

[B72] Zainal N. H. Newman M. G. (2024). A cross-lagged prospective network analysis of depression and anxiety and cognitive functioning components in midlife community adult women - CORRIGENDUM. *Psychol. Med.* 54:434. 10.1017/S0033291723003574 38037414 PMC10962314

[B73] Zhan Z. J. Huang H. Y. Xiao Y. H. Zhao Y. P. Cao X. Cai Z. C. (2024). Anxiety and depression in nasopharyngeal carcinoma patients and network analysis to identify central symptoms: a cross-sectional study from a high-incidence area. *Radiother. Oncol.* 197:110324. 10.1016/j.radonc.2024.110324 38735537

[B74] Zhang F. Lin C. Li X. Li M. Jia R. Guo X. (2023). The relationships between burnout, general wellbeing, and psychological detachment with turnover intention in Chinese nurses: a cross-sectional study. *Front. Public Health* 11:1216810. 10.3389/fpubh.2023.1216810 37546331 PMC10399590

[B75] Zhang J. Wang Y. Xu J. You H. Li Y. Liang Y. (2021). Prevalence of mental health problems and associated factors among front-line public health workers during the COVID-19 pandemic in China: an effort-reward imbalance model-informed study. *BMC Psychol.* 9:55. 10.1186/s40359-021-00563-0 33845895 PMC8040352

[B76] Zhen L. Wang G. Xu G. Xiao L. Feng L. Chen X. (2020). Evaluation of the paper and smartphone versions of the Quick Inventory of Depressive Symptomatology-Self-Report (QIDS-SR16) and the Patient Health Questionnaire-9 (PHQ-9) in depressed patients in China. *Neuropsychiatr. Dis. Treatm.* 16 993–1001. 10.2147/NDT.S241766 32368061 PMC7173799

[B77] Zung W. W. (1971). A rating instrument for anxiety disorders. *Psychosomatics* 12 371–379. 10.1016/S0033-3182(71)71479-0 5172928

[B78] Zyss T. Banach M. Zieba A. (2009). Akathisia–diagnosis, pathophysiology and therapy. *Psychiatr. Polska* 43 387–402. 20128247

