# Supplementary figures and images for: A network approach to understanding occupational psychological distress: linking depression, anxiety, and burnout among Chinese healthcare professionals
Source: Front Psychol. 2024 Dec 18;15:1474523. doi: 10.3389/fpsyg.2024.1474523 (PMC11690034; doi:10.3389/fpsyg.2024.1474523)

Average correlation with original sample

bridgeExpectedInfluence expectedInfluence

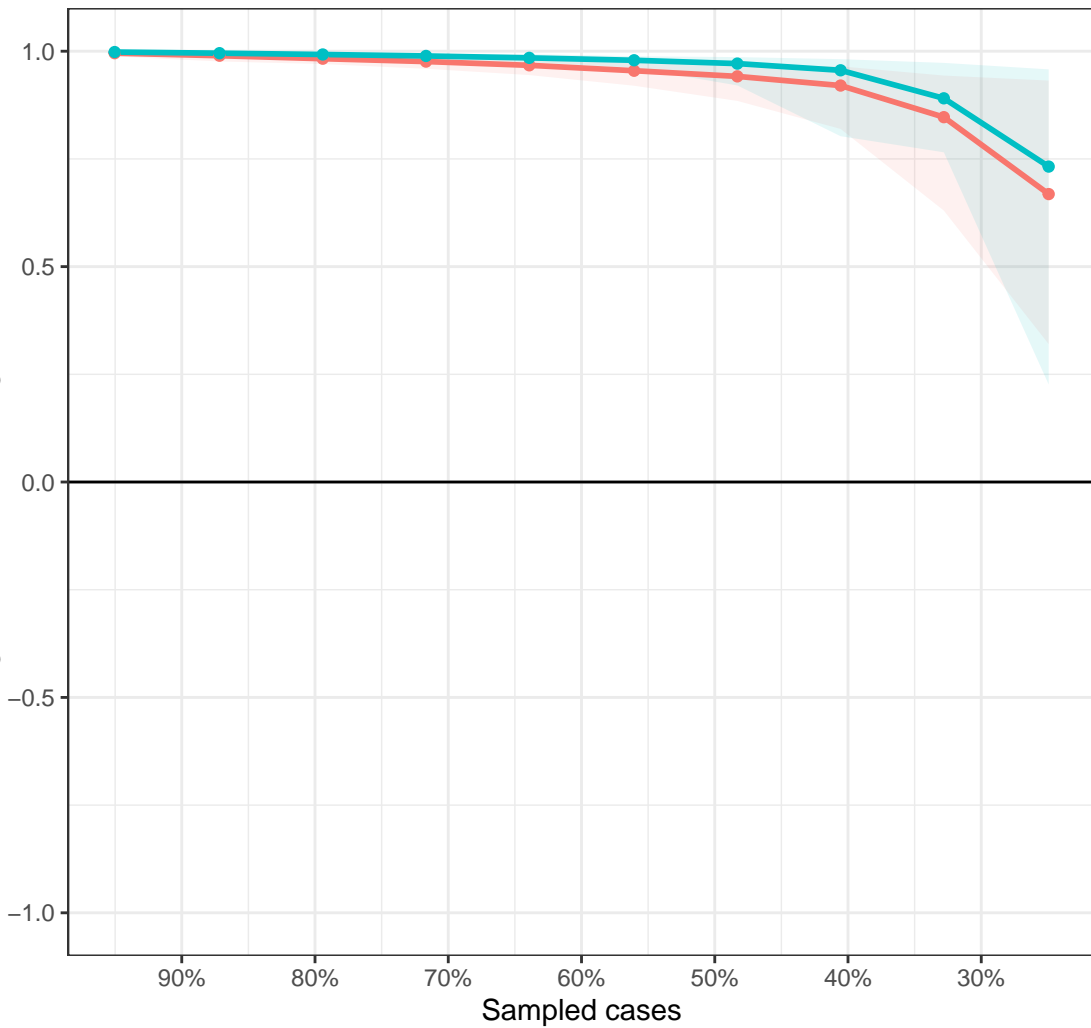

Supplement: Supplementary Figure 1 — Centrality stability for network. [file Data_Sheet_2.pdf]

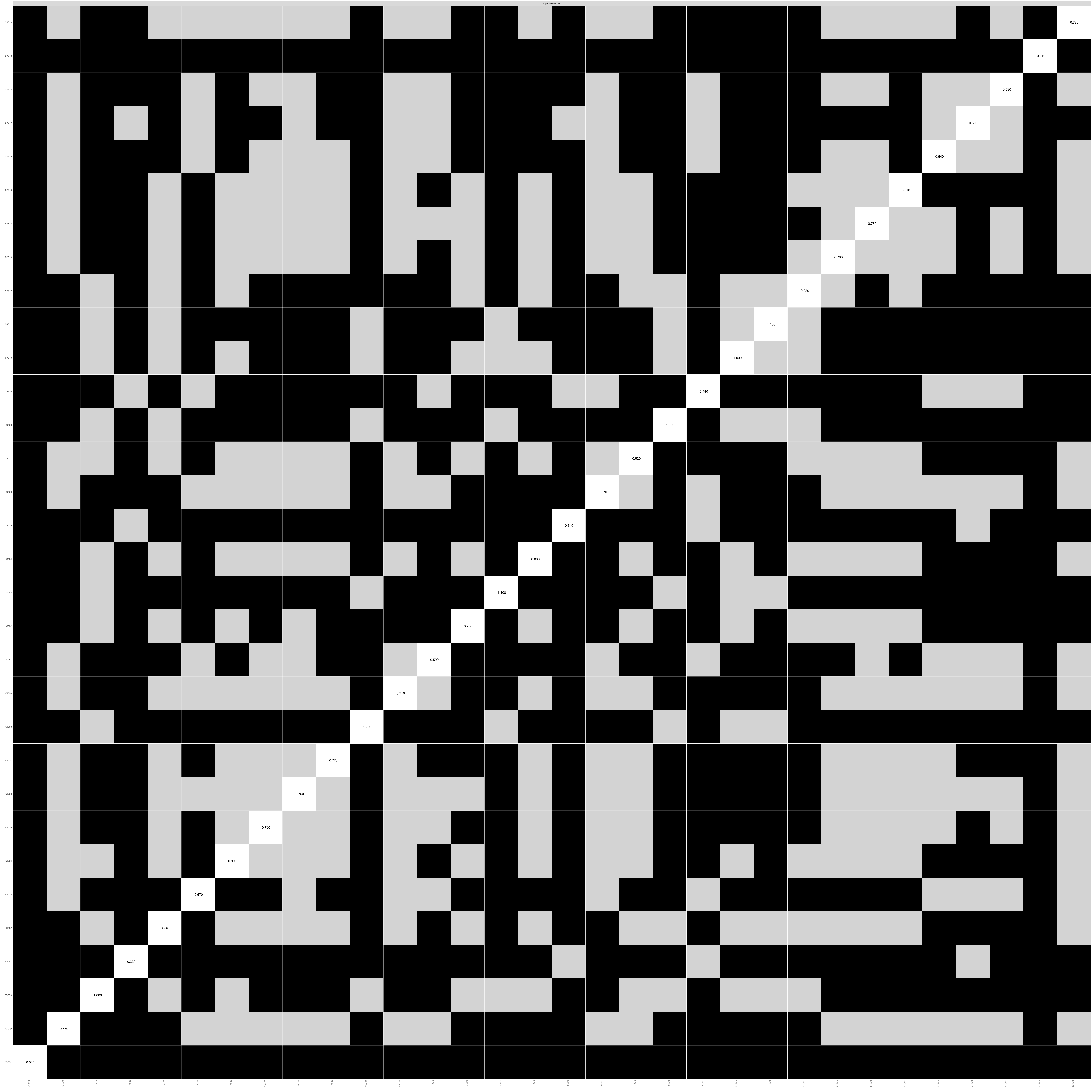

Supplement: Supplementary Figure 3 — Bootstrapped difference tests between expected influence. [file Data_Sheet_4.pdf]

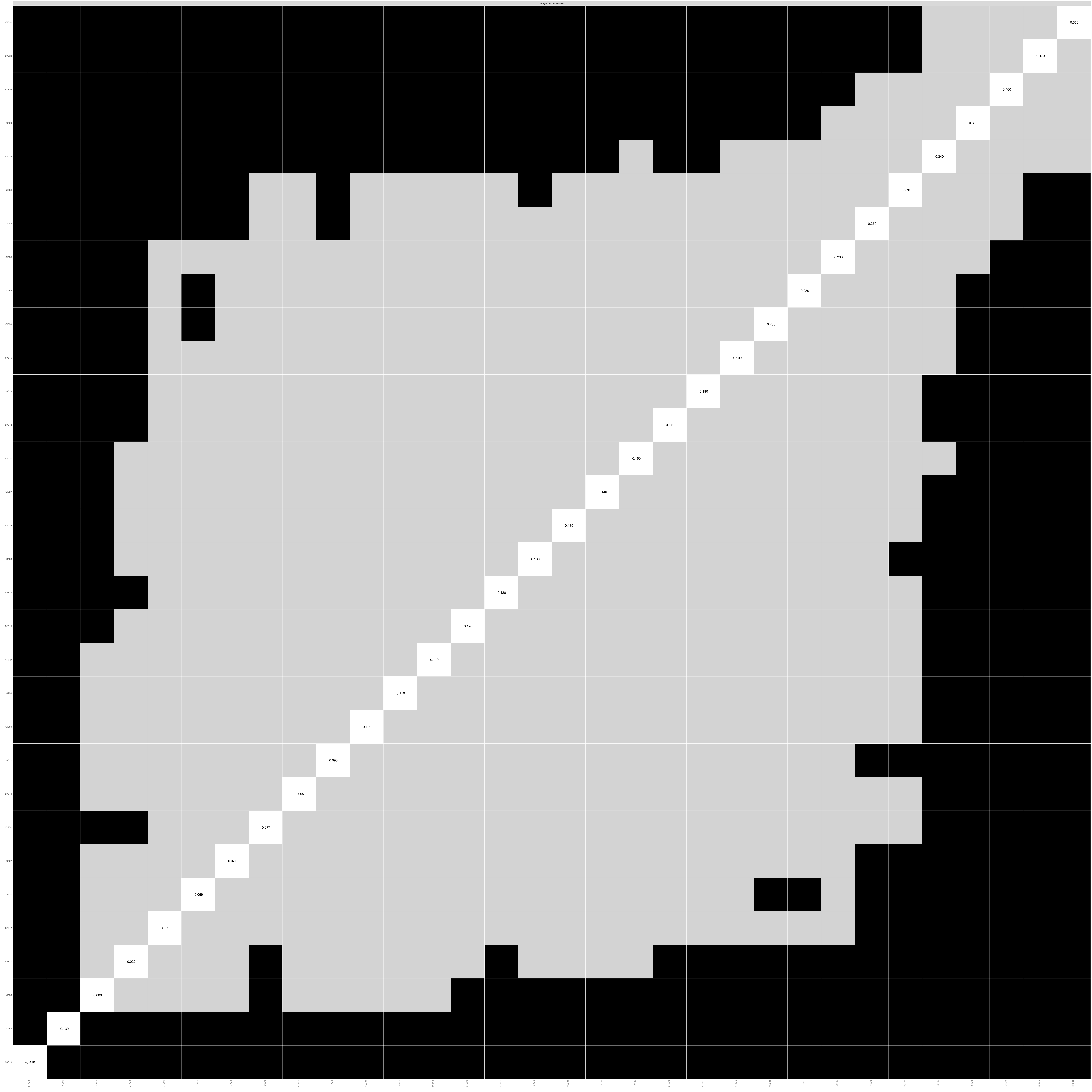

Supplement: Supplementary Figure 4 — Bootstrapped difference tests between bridge expected influence. [file Data_Sheet_5.pdf]
