# Supplementary material for: A network approach to understanding occupational psychological distress: linking depression, anxiety, and burnout among Chinese healthcare professionals
Source: Front Psychol. 2024 Dec 18;15:1474523. doi: 10.3389/fpsyg.2024.1474523 (PMC11690034; doi:10.3389/fpsyg.2024.1474523)
Supplement: Supplementary Figure 2 — Edge-weight accuracy for network. [file Data_Sheet_3.pdf]

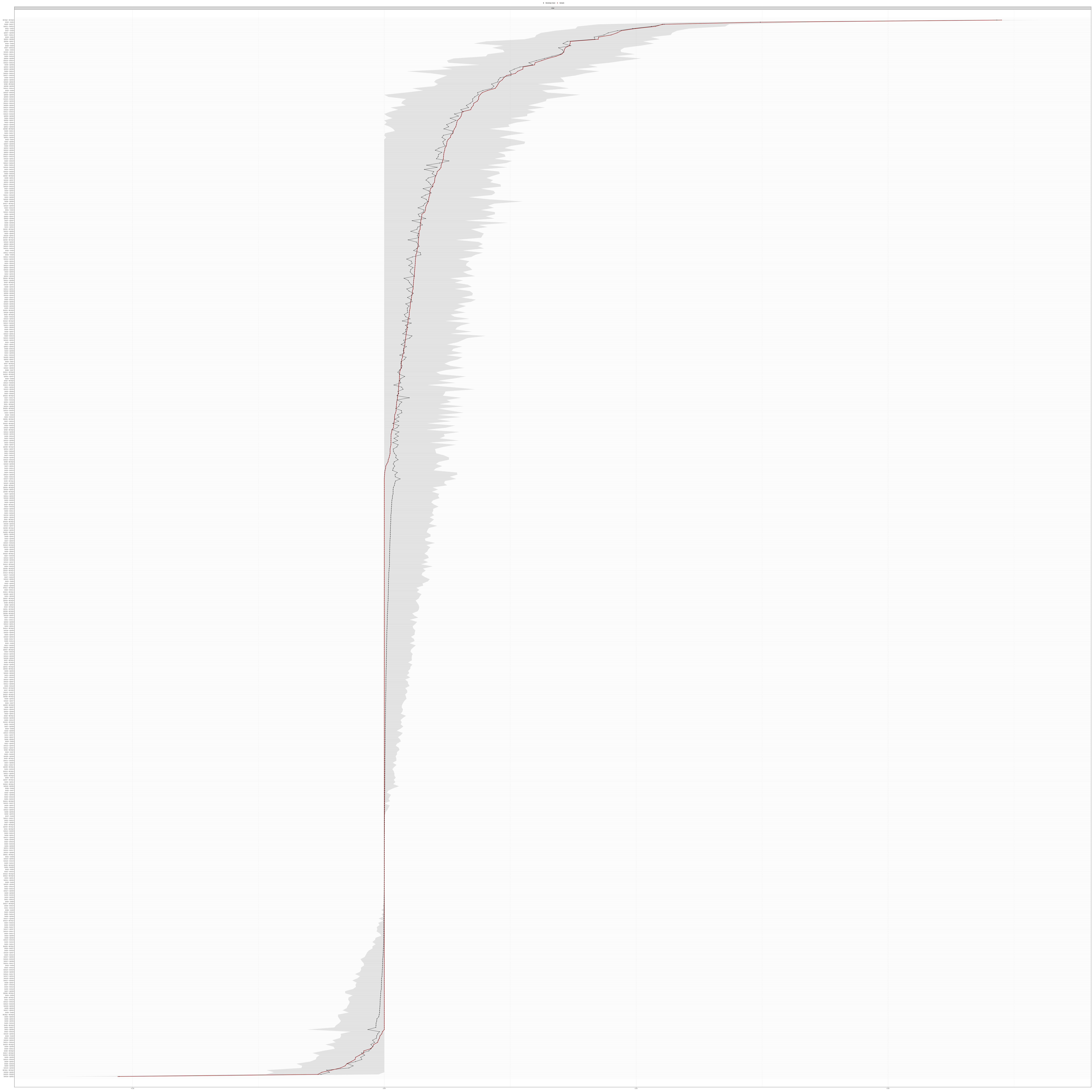

Bootstrap mean
Sample
edge
-0.25
0.00
0.25
0.50
